# The role of microglia in early neurodevelopment and the effects of maternal immune activation

**DOI:** 10.1007/s00281-024-01017-6

**Published:** 2024-07-11

**Authors:** L. J. M. Mastenbroek, S. M. Kooistra, B. J. L. Eggen, J. R. Prins

**Affiliations:** 1 Department of Obstetrics and Gynaecology, University Medical Center Groningen, University of Groningen, Groningen, the Netherlands; 2 Department of BioMedical Sciences, Section Molecular Neurobiology, University Medical Center Groningen, University of Groningen, Groningen, the Netherlands

**Keywords:** Neurodevelopment, Prenatal development, Immune system, Microglia, Maternal immune activation

## Abstract

Activation of the maternal immune system during gestation has been associated with an increased risk for neurodevelopmental disorders in the offspring, particularly schizophrenia and autism spectrum disorder. Microglia, the tissue-resident macrophages of the central nervous system, are implicated as potential mediators of this increased risk. Early in development, microglia start populating the embryonic central nervous system and in addition to their traditional role as immune responders under homeostatic conditions, microglia are also intricately involved in various early neurodevelopmental processes. The timing of immune activation may interfere with microglia functioning during early neurodevelopment, potentially leading to long-term consequences in postnatal life. In this review we will discuss the involvement of microglia in brain development during the prenatal and early postnatal stages of life, while also examining the effects of maternal immune activation on microglia and neurodevelopmental processes. Additionally, we discuss recent single cell RNA-sequencing studies focusing on microglia during prenatal development, and hypothesize how early life microglial priming, potentially through epigenetic reprogramming, may be related to neurodevelopmental disorders.

## Introduction

### Immune activation and neurodevelopment

The immune system plays an essential role during early development of the central nervous system (CNS). Maternal infection and activation of the maternal immune system have been associated with an increased risk for neurodevelopmental disorders (NDDs) in the still developing fetus [1]. In particular, autism spectrum disorder (ASD) [2] and schizophrenia (SCZ) [3, 4] are correlated to maternal immune activation (MIA) during pregnancy. The current evidence suggests that it is the maternal immune response towards a pathogen that can impact the developing brain and drives the increased risk for NDDs, and that the risk for NDDs is not pathogen-specific. Epidemiological data demonstrate how a range of pathogens triggering a MIA response are associated with an increased risk of NDDs in offspring [5], and this increased risk is not only limited to maternal infections. Other maternal immune stressors such as active atopic diseases like asthma [6] and auto-immune disorders [7] have also been associated with dysregulation of the immune system and an increased risk for NDDs in human offspring [8]. The development and manifestation of NDDs seems to be related to the timing of MIA, as evidenced by clinical and pre-clinical studies [9, 10]. Furthermore, animal studies demonstrate how administration of pro-inflammatory cytokines, specifically IL-6 or IL-17, are sufficient to cause NDD-like phenotypes in offspring, and conversely blocking cytokines during MIA induction can ameliorate these phenotypes [11, 12]. The ‘maternal immune activation hypothesis’ proposes that perturbations in immune interactions in utero during critical time frames of fetal development can drive neurodevelopmental disorders (NDDs).

### Microglia: the main tissue-resident macrophages of the CNS

**Fig. 1:**
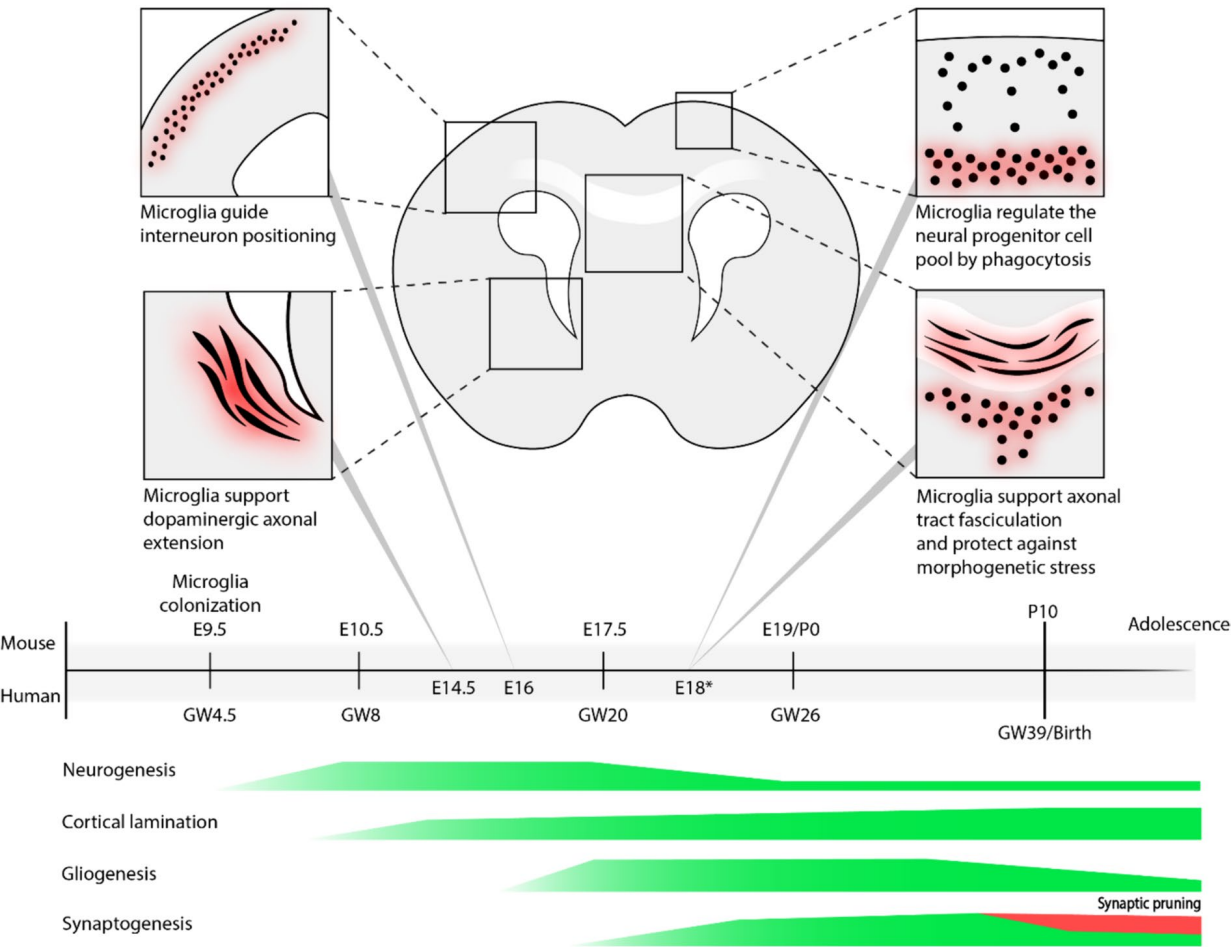
Timeline of microglial involvement in various early neurodevelopmental processes in the developing brain. Experimental evidence indicates a role for microglia in guiding the extension of dopaminergic axons and the positioning of interneuron populations. MIA results in perturbations in the patterning of somatostatin and parvalbumin (PV) expressing interneurons, which are persistent in postnatal life [22]. Microglia migrate to neurogenic zones and are involved in regulating the neural progenitor cell (NPC) pool through phagocytosis of NPCs [19–21]. Microglia localize to sites undergoing morphogenetic stress, and disturbing microglial function results in the formation of cavitary lesions specifically at these sites [26]. The timepoints on the timeline indicates at which embryonic days evidence of microglial involvement in neurodevelopmental processes was found, mostly based of mouse studies. Evidence for microglial localizing to, and regulating the neural progenitor cell pool was found in rats at E20, roughly corresponding to E18* in mice. The timeline roughly projects the timing of key developmental processes of the brain in both humans and mice

Microglia, the tissue-resident macrophages of the CNS, are considered a prime candidate for driving the increased risk of NDDs following immune activation during fetal development. As sentinels of the CNS, microglia continuously surveil their immediate surroundings through a combination of highly motile processes and an extensive repertoire of receptors collectively referred to as the microglial 'sensome’ [13–15]. In addition, microglia are able to rapidly react and adjust their phenotype accordingly [16, 17]. This enables microglia to act as immune responders to infection or injury and help maintain CNS homeostasis, fulfilling their primary physiological roles in the brain. When CNS homeostasis is compromised, microglia can respond by phagocytosing damaged neurons or aggregates and initiate an inflammatory response, thereby serving a neuroprotective role [18]. In the developing brain, microglia are also involved in various neurodevelopmental processes, including phagocytosing neural progenitor cells [19–21], guiding dopaminergic axonal extension, interneuron positioning [22], maintaining structural integrity, and selective pruning of supernumerary synapses [23–25] (Fig. 1). Disturbing microglial functioning during brain development can result in perturbations in the aforementioned processes.

Recent evidence suggest that already during early fetal development, microglia start to acquire a transcriptomic signature that resembles the adult microglia population marked by the increased expression of environmental- and immune sensing receptors [27]. An important implication of this is that microglia possibly already acquire their mature immune sensing properties at early fetal developmental stages, likely making them sensitive to immune activation during critical moments of fetal development. Indeed, a growing body of evidence consisting mostly of animal studies demonstrates that MIA can evoke a microglial response in the developing fetal brain. In parallel to the activation of microglia, MIA also results in neuropathological- and behavioural hallmarks resembling aspects of NDDs [28, 29]. Interestingly, microglia emerge in the brain early in development, before peak cortical neurogenesis and neuronal migration, and prior to the emergence of other glial cells, astrocytes and oligodendrocytes [30, 31]. So on top of their role as the primary immune responders of the CNS, they also uniquely inhabit the fetal brain during a key developmental time frame. Seeing how a growing body of evidence suggest that microglia are intricately involved in early neurodevelopmental processes, the timing of MIA might perturb microglial functioning and disrupt proper neural circuit formation thereby driving the increased risk for NDDs in the offspring.

In this review, we will discuss the physiological role of microglia in prenatal and early postnatal neurodevelopment, and examine the experimental evidence on the impact of MIA on microglia in relation to these neurodevelopmental processes. Additionally, we highlight the findings of recent scRNA-seq studies aimed at studying microglial heterogeneity during prenatal development, and hypothesize how the development of a MIA-induced innate immune memory, potentially governed by epigenetic reprogramming, might be linked to neurodevelopmental disorders.

## The physiological role of microglia during early neurodevelopment

### Microglial origins

Microglial cells have a unique ontogeny compared to the other major CNS cell types which derive from the neuroepithelial cells forming the wall of the neural tube. Instead, microglia originate in the yolk sac deriving from haemopoietic precursors and will colonize the developing CNS in distinct waves, prior to the closure of the blood–brain barrier [32]. The expression and activation of colony stimulating factor 1 receptor (CSF1R) is essential for the differentiation and development of the microglial cell lineage [32], and, in humans, loss of CSF1R due to a homozygous mutation can lead to a complete absence of microglia and structural brain malformations, epilepsy and neurodevelopmental regression [33]. In mice, primitive microglia start populating the developing CNS at embryonic day 9.5 (E9.5) [32], and this is observed at 4.5 gestational weeks (GWs) in humans [34, 35]. The emergence of microglial cells is commonly defined by the expression of ionized calcium binding adaptor molecule 1 (IBA1) in myeloid cells. Transmembrane protein 119 (TMEM119) can also be used to identify microglial cells, however, TMEM119 only stains a subset of homeostatic microglia. Microglia display a heterogenous pattern of colonization and distribution during development [36]. In the cortex, IBA1 + microglia have an initial increase in proliferation around 6 GWs, followed by a massive first wave of proliferative expansion peaking at 8 GWs [35]. Next, the expanded microglial cell pool shrinks through apoptosis, followed by a second less significant wave of proliferative expansion at 16 GWs until cell density drops again and reaches an equilibrium around 28 GWs [35]. Eventually, microglia will form a self-sustaining tissue resident macrophage population and act as the first responders to pathogenic intruders and tissue damage, thereby help in maintaining CNS homeostasis [37]. Notably, microglia already start populating the human embryonic CNS preceding peak neurogenesis and prior to the emergence of other glial cells, astrocytes and oligodendrocyte lineage cells [30, 31](Fig. 1). Moreover, in the developing embryonic cortex, microglia preferentially localize at sites important for neurogenesis [14, 20], hinting at microglial involvement in early neurodevelopment.

### Microglia and prenatal neurodevelopment

Microglia are regulators of neurogenesis in the prenatal brain (Fig. 1). Early in the developing cortex, neural progenitor cells (NPCs) in the ventricular zone (VZ) and subventricular zone (SVZ) are proliferating and differentiating into radial glial cells. Radial glia asymmetrically divide into neuronal cells while also serving as scaffolding for newly formed maturing neurons to radially migrate to their correct cortical layers [38]. Only later in development, around 20 GWs in humans, radial glia begin producing the other CNS resident glial cells, astrocytes and oligodendrocytes [30, 31]. Both human and animal studies demonstrate how microglia are attracted and localized to sites of enhanced neurogenesis during early fetal neurodevelopment, in particular the cortical proliferative zones (the ventricular zone (VZ) and subventricular zone (SVZ)) [19–21]. Microglia migrate to sites of dead- or dying cells and can be directed through the expression of chemokines (CXCL12) by cortical progenitors [19]. Microglia have been shown to guide normal brain development through their phagocytosis of dead or dying cells [39], a feature seen in both the prenatal and adult CNS [40]. Notably, at these highly neurogenic sites of the VZ/SVZ, microglia closely interact with and phagocytose neural precursor cells (NPCs) which do not all necessarily undergo apoptosis [20, 41]. Thus, microglia can actively deplete viable NPCs through selective processes other than the apoptotic pathway and regulate the pool of NPCs. Additionally, early in the postnatal brain (P3), microglia have been demonstrated to promote neural cell death [42], and modulating an increase or decrease in microglial activation was able to decrease or increase the NPC pool, respectively [20]. Conversely, microglia can also promote the migration and differentiation of NPCs [43, 44], and in the early postnatal brain of rats it was found that microglia can promote proliferation of neuronal and oligodendrocyte progenitors, possibly through the release of inflammatory cytokines [45].

Microglia also guide axonal tract organization and positioning of interneurons. During development, microglia closely associate with dopaminergic axonal tracts in the cortex, and perturbing microglial functioning through immune activation resulted in reduced extension of dopaminergic axons and the positioning of interneuron populations, including parvalbumin (PV) expressing interneurons [22]. This is particularly interesting, since dysfunction of the dopaminergic and PV interneuron circuits have both been strongly implicated in neurodevelopmental disorders such as SCZ [46]. Additionally, microglia associate with the corpus callosum, a structure consisting of organized axonal bundles connecting the left and right brain hemisphere [47]. Perturbing microglial functioning resulted in disorganization of the dorsal callosal axons [47]. scRNA-seq data identified a specific subcluster of microglia, seemingly in an activated state based on their transcriptional and morphological signature, that tightly associate with the subcortical axon tracts of the corpus callosum and the cerebellum in the early postnatal brains of mice [48]. A similar subpopulation of microglia was identified in the rodent and human prenatal brain that tightly localize to sites bordering the cortex undergoing morphogenetic stress, following the natural progression of prenatal brain development [26]. Transiently depleting microglia in mice results in cavitary lesions at these sites, suggesting a protective role of microglia maintaining the integrity of CNS structures undergoing morphogenetic stress [26]. In summary, experimental evidence demonstrates that during the critical window of prenatal CNS development, microglia are involved in early neurodevelopmental processes, including neurogenesis, dopaminergic axonal extension, interneuron positioning, maintaining CNS structural integrity, and disturbing microglial functioning can disrupt these processes.

### Microglia and early postnatal neurodevelopment

Microglia continue to support neurogenesis and neural circuit maturation in the postnatal brain. In the hippocampus, microglia engulf and phagocytose apoptotic neurons while not necessarily being in an activated state [49]. Microglia also phagocytose oligodendrocyte progenitor cells (OPCs) and pre-myelinating oligodendrocytes [50], and a subset of CD11c + microglia play a role in oligodendrocyte maturation and myelination in the early postnatal brain [51]. Furthermore, microglia start specialising in regulating synaptic densities. In the developing brain, there is an initial overproduction of synapses, followed by the elimination of excess and weak synapses, which continues until adolescence. This process is essential for the refinement of neural circuits and cognitive development. In their resting state, microglia frequently contact nearby synapses in an activity-dependent manner [52, 53] and can remodel synaptic connections accordingly [24]. Early in postnatal development during peak synaptogenesis, microglia actively engulf and eliminate synapses, a process called ‘synaptic pruning’ [23]. Microglia selectively prune synapses of less active neurons [24, 25], thereby microglia are likely assisting in the remodelling for efficient neural connectivity. In mice, it was demonstrated that microglial depletion in the early postnatal period (P4) results in a temporary increase in synaptic connections from both excitatory and inhibitory neurons, including PV + and somatostatin + interneurons, projecting unto excitatory neurons [54]. A subpopulation of GABA-responsive microglia was found to remodel inhibitory circuits by preferentially engulfing and pruning inhibitory synapses [54]. Inhibiting microglial-neural interactions by knocking out either the neuronally expressed fractalkine (*CX3CL1*), or the fractalkine receptor (*CX3CR1*) primarily expressed by microglia results in inhibition of synaptic elimination on neurons downstream after sensory lesioning [25]. Additionally, deletion of *CX3CR1* in mice resulted in a transient increase in synaptic density and a delay in neural circuit maturation [23]. The exact mechanism underlying microglial-dependent synaptic elimination is not completely understood. In mice, it was demonstrated that synaptic pruning during CNS development is also dependent on astrocyte-microglial interactions through cytokine signalling, indicating that other glial cells are also involved in regulating synaptic density [55]. Moreover, while studies conclude how microglia completely engulf and phagocytose synaptic material [23], a form of incomplete engulfment called ‘trogocytosis’-or nibbling- has also been described leading to the elimination of pre-synaptic material [56]. On the other hand, microglia can also promote the formation of dendritic spines in the early postnatal brain [57] and during learning-dependent remodelling of dendritic spines in mice [58]. Overall, microglia play an active role in the formation and maturation of functional synaptic connectivity during development.

## Microglial heterogeneity throughout prenatal neurodevelopment from a scRNA-seq perspective

Recently, the advancement of single cell techniques has allowed for unbiased analysis of the transcriptomic changes in microglia during development. Using scRNA-seq, it was demonstrated that during normal development, human fetal microglia initially form a highly heterogeneous pool of cells compared to the adult population [27]. Moreover, human [27, 59] and animal [48, 60] scRNA-seq studies demonstrate how distinct microglial transcriptomic phenotypes are observed at specific moments and brain regions during fetal development. This suggests that in early life, unique subpopulations of microglia acquire specialised transcriptomic profiles likely associated with functional consequences, coordinated by their spatial–temporal context. A set of microglial clusters can be identified enriched in neuronal markers, consistent with the physiological role of microglia phagocytosing neuronal cells and synapses [27, 59]. A set of ‘immune-enriched’ microglial clusters can also be found, marked by the increased expression of genes related to inflammation [27, 59]. The neuronal-enriched clusters were found to become region-specific and predominantly emerged from 8–18 GWs [59]. Clusters of immune-enriched microglia, were found throughout the fetal brain, as well as region specific clusters arising only in later stages of development [27, 59]. On the other hand, clusters of microglia enriched in proliferation markers can be found throughout development, displaying no region-specific distributions, seemingly indicative of their collective proliferative potential [59]. The functional significance of the individual clusters is not yet clear. A striking finding across the single-cell studies focusing on fetal microglia, is the increased expression of genes associated with ‘disease associated microglia’ (DAM), including *CD11c*, *SPP1*, *APOC1*, *APOE*, *CLEC7A*, and *GPNMB* [27, 59, 61]. This suggests that in the context of neurodegenerative disease, the early developmental microglial transcriptomic programming is rekindled [62]. In mice, microglial subsets have previously been described closely associating with axonal tracts of the corpus callosum and proliferating areas coined ‘axonal tract-associated microglia (ATM)’ [63] and ‘proliferation-associated microglia (PAM)’ [64], respectively, which share a DAM-like signature. Disturbing microglial functioning was found to result in the disorganization of axonal bundles in the corpus callosum [47]. ATM were originally identified in the early postnatal brain of mice but ATM-like cells are now also found accumulating at specific sites bordering the cortex, both in the prenatal rodent and human CNS [26]. Transiently deleting microglia resulted in cavitary lesions specifically at these undergoing morphogenetic stress, suggestive of their role in maintaining structural integrity [26]. These results indicate that microglial subtypes sharing a DAM phenotype have a reactive phenotype and are involved in specific neurodevelopmental processes. Still, the functional significance of DAM-like phenotypes during early development is not yet clear, and the transient emergence of DAM-like cells in various contexts might indicate a generalized microglial response towards different environmental stimuli [65].

Progressively during fetal development, microglia start to acquire a more homeostatic and homogenous transcriptomic signature that is characterized by the expression of environmental- and immune-sensing receptors and bear more resemblance to the adult microglial population [27]. Notably, in humans already at early fetal developmental stages (GW12-13) microglia start to acquire mature immune sensing properties, possibly making them more susceptible to immune activation. The microglial transcriptome going from a heterogenous, proliferative phenotype to a more homogenous, homeostatic and immune-sensing phenotype during development is also seen in mice, though the timing is different compared to humans [63, 66, 67]. A recent scRNA-seq paper in mice, demonstrated that MIA initially leads to transcriptomic changes in fetal microglia, including the differential expression of genes related to proliferation and immune activation [68]. There were no changes in the proportions of clusters or the identification of unique clusters shortly after stimulation and in juveniles (P14) [68]. However, they did find a small subset of genes differentially expressed across all microglial clusters, and remained differentially expressed in juveniles [68]. In mice, MIA exposure causes the fetal microglial transcriptome to become more similar to the adult population [66]. This indicates that immune activation causes microglia to progress earlier to their more mature, immune-reactive phenotype [66]. The observation of accelerated microglial transcriptional maturation following immune stimulation is also seen when adolescent mice are challenged, specifically in males [67]. Potentially, the timing of the MIA-induced shift in microglial molecular machinery to a more ‘advanced’ phenotype during prental neurodevelopment might disrupt early microglial functioning and cause disruptions in the still developing CNS. In line with this thesis, scRNA-seq of human fetal CNS tissue revealed a population of ‘immune-related microglia’ which spontaneously exited their initially acquired resting status at GW23 through the downregulation of homeostatic genes (*TMEM119*, *P2RY12/13*, and *CX3CR1*) and upregulated expression of *CD68* [59]. Perhaps, this switch in immune phenotype follows the natural step-wise maturation process of microglia, and immune activation through external stimuli perturbs this step-wise maturation process and, consequently, disrupts microglial functioning. The transition from a homeostatic to an active transcriptomic signature is conserved in mice; however, in mice, this transition occurs early postnatal life (P2), highlighting a difference in microglial development between humans and mice [59]. However, this is still consistent considering that the neurodevelopmental processes happening predominantly during late gestation in humans continue through the postnatal period in rodents [69].

One study identified a rare population of CD4 + T cells in the mouse and human brain, and found that presence of the CD4 + T cell population was required for immature and developing microglia to complete their fetal-to-adult transcriptional maturation in mice [70], showcasing a potential role for the peripheral immune system in microglial development. The immature transcriptional profile of microglia showed a relatively lower expression of genes involved in synaptic pruning and was found alongside increased synaptic densities of cortical pyramidal neurons, implicating perturbations in microglial functioning and pruning [70]. Microglial crosstalk with T cells has mainly been studied in the context of neuropathological conditions, but seeing the involvement of the peripheral immune system in microglial development warrants further investigation, despite the scarcity of peripheral immune cells in the normal developing brain.

## The effect of MIA on microglia and neurodevelopment

Emerging evidence demonstrates how microglial-neural crosstalk is intricately involved in shaping the complex CNS cytoarchitecture by guiding neurogenesis, neural wiring and synaptic remodelling early in development. Possibly, by perturbing microglial-neural crosstalk through exposing immune sensitive microglia to MIA during the critical timeframe of fetal neurodevelopment can disrupt early neurodevelopment, and drive the increased risk for NDDs.

### Animal models of MIA recapitulate aspects of NDDs

Animal models of MIA recapitulate behavioural- and neuropathological aspects seen in NDDs, thereby providing an invaluable tool to study the effects of MIA on neurodevelopment. Commonly, these animal models of MIA use injections of either i) a synthetic analogue of double-stranded RNA (Poly I:C) or ii) a component of the bacterial cell wall called lipopolysaccharide (LPS) to elicit an immune response [71]. Poly I:C is recognized by toll-like receptor 3, member of a class of transmembrane proteins with a role in innate immunity whereas LPS is mainly recognized by toll-like receptor 4 [71]. In animal models, MIA results in repetitive behaviour and abnormal social interactions corresponding to ASD-like behaviour [28]. Furthermore, MIA induction results in behavioural alterations mimicking aspects characteristic of SCZ, including increased locomotor activity upon stimulation with amphetamine, as well as deficits in sensorimotor gating [28]. Several structural and functional brain alterations are observed in the brain following MIA including changes in dopaminergic circuits which is a neuropathological hallmark strongly associated with SCZ in humans [28, 29]. In line with clinical data, the timing of MIA in animal models seems to be an important determinant in the manifestation of the behavioural and neuropathological phenotype seen in the offspring [72]. We can also draw parallels with clinical observations of sex-specific differences in NDD manifestation and the behavioural and neuropathological phenotypes in animal studies [73].

### The effect of MIA on microglial density and ‘activation’

Numerous animal studies have described changes in microglial cell number and increased microglial ‘activation’ in offspring following MIA exposure, however these findings are contrasted by studies reporting no change in microglial density or activation [28]. Likely, these inconsistencies are the result of methodological differences, such as strategies for inducing MIA (i.e., LPS or Poly I:C; timing and dosing of MIA), readout of microglial ‘activation’ markers, and the timing of readouts [74]. It is also important to consider the complex microglial heterogeneity in their spatio-temporal context, especially during early neurodevelopment as highlighted by scRNA-seq studies on fetal CNS tissue [27, 59, 63, 66]. Microglial density and single activation markers cannot capture the complete spectrum of microglial ‘activation’ states and functional responses. The sum of these factors makes it difficult to compare and interpret the literature studying the effect of MIA and microglial involvement. Nevertheless, numerous studies support that fetal microglia are activated and respond to MIA, and this is backed up by studies noting an increase in the microglial gene expression of pro-inflammatory cytokines following MIA [75–77]. Furthermore, MIA induced changes in the microglial transcriptome and phagocytic function have been observed in adult microglia [78].

### MIA-induced effects on the neuronal level

MIA leads to microglial activation, disrupts the transcriptome of cortical neuronal cell types, and leads to perturbations in neurogenesis and interneuron dysfunction. MIA induction in mice causes lasting changes in the transcriptomic signature of the cortex, as well as changes in the composition of NPCs, cortical lamination and glial cells [79]. In the prenatal CNS, microglia most robustly express receptors responsive to MIA-related immune factors, including receptors for IL-6 and IL-17 which have been causally linked to MIA and its neurobehavioral phenotypes [11, 12, 68]. In the fetal mouse brain, MIA was found to activate the integrated stress response (ISR) leading to an overall reduction in protein synthesis in cortical neuronal cell types and behavioural phenotype in males specifically [80]. This mechanism was found to be dependent on IL-17a signalling, suggesting that microglia are responsible for mediating this effect [80]. In line with this study, the MIA-induced transcriptomic changes in neuronal cell types could be reduced when genetically ablating microglia in *Csf1r* null mouse [68], although the absence of microglia in itself likely has an effect on the cortical transcriptome.

In the prenatal brain, microglia preferentially cluster at the highly neurogenic zones of the VZ/SVZ and were demonstrated to phagocytose NPCs [20]. Modulating microglial activation through MIA decreases the number of NPCs, while minocycline administration or microglial depletion increased the number of NPCs, without affecting proliferation, consistent with increased phagocytosis of NPCs by microglia following MIA [20]. MIA was also shown to lead to a decrease in the total number of PV interneurons in male rats [77]. Other studies also found a decrease in PV interneurons after MIA [81–83], as well as selective dysfunction of PV interneuron GABAergic transmission projecting onto the pyramidal cells in the mPFC in adult offspring [82]. Optogenetically silencing PV interneurons is able to recapitulate the anxiety-like behaviour seen after MIA [82]. To examine the role of microglia in the dysfunctional PV interneuron network following MIA, it was demonstrated that the decreased inhibitory drive from PV interneurons in adults (P60) can been seen after both MIA and upon microglial depletion using CSF1R inhibition [84]. Surprisingly, there was an initial increase in the inhibitory drive in juveniles at postnatal day 20 (P20), which might reflect improper development of the PV interneurons [84]. Using a different approach, it has been shown how MIA results in downregulation of expression of *GPR56* in fetal microglia, through maternal IL-17a signalling [85]. Deletion of *GPR56* in mice inhibits the proliferation of neural progenitors committed to forming PV interneurons and mimics the decreased number of PV interneurons and behavioural phenotype seen after MIA. Restoring *GPR56* expression after MIA was able to partly rescue PV interneuron deficits and autism-like behaviour in mice at P21 [85]. In another study looking at the hippocampus of adult offspring following MIA exposure, found that minocycline treatment (P21-P56), a known inhibitor of microglia, was able to reverse the behavioural deficits, neuroinflammation, microglial ‘activation’ and the decrease in PV interneurons, as well as rescue the MIA induced functional alterations of PV interneuron activity and GABAergic transmission [86]. Increasing microglial expression of arginase 1 (*ARG1*), which is involved in regulating microglia activation and neuroinflammation, was able to ameliorate MIA-induced behavioural- and PV interneuron deficits, whereas ablation of microglial *ARG1* expression prevented the protective effects of minocycline treatment [86]. Overall, modulating microglia functioning seems to recapitulate the MIA induced deficits in the PV interneuron network. In vitro data shows how an induced pluripotent stem cell (iPSC) derived co-culture of activated microglia and developmental cortical interneurons results in disruptions of interneuron metabolic pathways, arborization and synapse formation, with persistent metabolic dysregulation in the SCZ-derived cortical interneurons but not healthy controls [87]. In summary, there is evidence supporting that the effect of MIA on the cortical neuronal cell types, including PV interneurons, may be at least partially mediated through microglia.

### MIA-induced effects on the synaptic level

Microglia are also involved in regulating the formation of neural connectivity on the level of individual synapses, and alterations in the densities of synapses have been observed in offspring following MIA induction. Especially during adolescence and early adulthood there is extensive elimination of supernumerary synapses, a process in which microglia are intricately involved through synaptic ‘pruning’, a process dependent on neural activity and the complement system [23, 24]. In 1982, Feinberg already postulated that alterations in synaptic composition due to dysregulated synaptic pruning might underly NDDs like SCZ, which is now referred to as the ‘synaptic hypothesis’ [88, 89]. Meta-analyses of post-mortem studies looking at the brains of SCZ patients found decreased pre-synaptic and post-synaptic markers, suggestive of alterations in synaptic densities that are supported by electron microscopy studies [89]. A post-mortem study of the brains from individuals aged 4 to 22 years old with ASD found that the expression of genes involved in synaptic function, GABAergic signalling, axon guidance, and neuronal migration were dysregulated and coincided with ASD annotated risk genes [90]. Specifically, microglia demonstrated enrichment in genes associated with activation, as well as transcriptional factors implicated in developmental processes, which exhibited correlations with clinical severity [90]. Still, post-mortem studies can be subject to confounding factors like the use of medication, lifestyle, brain region investigated, and disease state at time of a donors death. Additional evidence suggesting disrupted synaptic pruning in neurodevelopmental disorders (NDDs) involves genetic variations in complement component 4 (C4), which is part of a complement protein cascade tagging synapses for elimination by microglia. Genetic variations of C4 have been strongly linked with an increased risk for SCZ, and an increased expression of C4 has been found in the brain of SCZ patients [91]. Differential gene expression analysis of the fetal rat brain shortly after MIA showed changes in expression of genes related to neurogenesis, axonal guidance and synaptogenesis [92]. Possibly, MIA can perturb microglial-dependent synaptic pruning and affect proper neural network maturation.

Animal studies report MIA-induced alterations in microglial-synaptic contacting, dysregulated pruning and changes in synaptic densities in offspring. Using their motile processes, microglia frequently contact synapses in an activity-dependent manner [52, 53] and can remodel synaptic connections accordingly [24]. In acutely isolated brain slices from MIA-induced mice, alterations were found in the process motility of fetal and adult microglia [93]. Changes in microglial process motility could be also be observed in non-MIA-exposed brain slices through IL-6 stimulation [93]. Additionally, MIA exposure results in an increased expression of microglial genes related to synaptogenesis, neuronal cell adhesion/filopodia formation, and an increase in microglial interactions with excitatory pre-synaptic structures, enhanced filopodia formation and spine density in layer V at P60, as well as changes in their neurophysiological properties [94]. This suggests that MIA induces changes in microglial-synaptic interactions and/or pruning. Microglial repopulation using CSF1R inhibition could ameliorate the behavioural phenotype and reverse the increase in microglial-neuronal interactions [94].

MIA results in increased synaptic densities, possibly through inhibition of microglial-neural interactions. After MIA induction, the spinal density of granule cells in the hippocampus was increased at P15, a critical period for synaptic pruning, but only in male mice [95]. In parallel, there was a male-specific decrease in the expression of *CX3CR1* on the mRNA [95] and protein level [96]. CX3CR1 is primarily expressed by microglia in the CNS and knockout of *CX3CR1*, a mediator in microglial-neural interactions and synaptic pruning, results in a transient increase in spinal density as well as ASD-like behavioural deficits [23, 25]. Possibly, reduced microglial expression of CX3CR1 is limiting microglia-neural interactions and, consequently, inhibiting synaptic pruning ultimately leading to the increase in synaptic density seen early in postnatal life. Chamera et al., 2020 also noted changes in the mRNA and protein expression of the *CX3CL1*-*CX3CR1* and *CD200*-*CD200R* pathways in the hippocampus and cortex following MIA, depending on the use of LPS or Poly I:C, but neither immunostimulant affected the co-localization of CX3CL1-CX3CR1 and CD200-CD200R at P7 [97].

In contrast, other studies demonstrate how MIA results in decreased synaptic densities. After MIA, activation of microglia was seen alongside decreased protein levels of pre- and post-synaptic makers and ultrastructural changes in the cortex of adult (P52-P54) rats [98]. In mice, MIA resulted in a decrease in pre-synaptic density in the hippocampus and dentate gyrus, observed only in adult mice [99]. When looking at the post-synaptic density, a decrease in was noted both in pubescent and adult mice [99]. In the cortex of adult rat offspring, MIA resulted in an increased neuro-inflammatory profile, as measured by an increase in the expression of inflammatory cytokines, and co-localization of Il-1β with microglia, and a decrease in pre-synaptic vesicles and synaptic density [98]. In a different MIA model, a combination of air pollutants and resource deprivation was used to induce stress in mice resulting in a maternal pro-inflammatory response similar to the Poly and I:C model [100]. On top of the aberrant social behaviour in male offspring, gene expression analysis showed how most differentially expressed genes were enriched in microglia, including synapse genes [100]. When examining the thalamicocortical circuits, this MIA model resulted in an initial male-specific overgrowth of synapses at P8, followed by a reduction in thalamicocortical connections at P15 persistent at adulthood [100]. Compared to no stress exposure, microglia in MIA-induced mice offspring were found to engulf less synapses and were less phagocytic at P10 [100]. Another study also found a decrease in dendritic spine density and less extensive dendritic branches in adulthood in the granule cells of the dentate gyrus and layer II/III pyramidal mPFC neurons [101]. A SCZ patient-derived co-culture of microglia and synaptosomes revealed that SCZ-derived microglia displayed a higher uptake of synaptic material compared healthy control derived microglia, demonstrating a link with SCZ genetic risk to altered synaptic pruning [102]. In summary, there is strong evidence demonstrating that MIA causes alterations in synaptic/spine densities and microglial-synapse interactions, suggesting dysfunctional microglia-dependent synaptic pruning. This is further supported by studies demonstrating that microglial repopulation using CSF1R inhibition can ameliorate MIA-induced effects on spine densities and their electrophysiological properties [94]. Interestingly, there is evidence pointing towards a time-dependent effect of MIA, with studies showing an initial increase in synaptic densities early in post-natal life followed by a decrease in synaptic density in the adult brain consistent with post-mortem findings in human SCZ patients [89, 103]. However, whether these changes are truly time-dependent remains elusive since several other studies report opposite results in synaptic densities at similar timings, although these findings might be brain region dependent [104].

## MIA-induced reprogramming of microglia and a possible role for epigenetics

One of the questions that remains is how stimulation of microglia in the fetal brain can have such long-lasting consequences. Microglia have a slow turnover compared to other hematopoietic cells and have a long lifespan that can extend to more than 20 years [105], and so early life-programming can potentially have persistent and adverse effects later in postnatal life. Indeed, MIA has been shown to cause changes in the microglial transcriptome that persist into adulthood in the offspring, although the effect seems relatively small [68, 78]. A recent fate-tracing study investigated the dynamics of DAM-like microglia using a model of stroke and resolution in neonatal (P6) and juvenile (P30) mice [106]. The authors found that DAM-like microglia induced by stroke in neonatal mice were able to regain their homeostatic phenotype following stroke resolution [106]. In contrast, when stroke was induced in juveniles, nearly all DAM-like microglia were eliminated while the few remaining cells maintained their DAM-like phenotype [106]. This demonstrates that at earlier developmental time-points, activated microglia can reintegrate back into the CNS parenchyma as seemingly homeostatic microglia. However, when re-exposed to an immune trigger (LPS), the reintegrated ‘homeostatic’ microglia show an enhanced inflammatory response [106]. This altered response to a secondary immune stimulus is reminiscent of innate immune memory as observed in adult animals [107, 108]. This is consistent with MIA models, where prenatal immune exposure similarly leads to an ‘innate immune memory’ and an altered inflammatory response by microglia in postnatal life [75, 109]. However, instead of an enhanced immune response, LPS re-exposure in adulthood results in a diminished inflammatory response, as measured by a decrease in microglial expression of pro-inflammatory cytokines, in whole brain of mice [75]. There was a region-specific increased inflammatory response in the hippocampus [75]. In line with the previous study, a diminished microglial immune response was also found in the frontal cortex and striatum, as measured by the downregulation of genes involved in immune pathways, microglial morphology and lysosomal content [109]. These results point towards microglia undergoing ‘immune tolerance’ when exposed to immune activation in prenatal life, although this effect shows brain region-specific changes since hippocampal microglia results in ‘immune training’. Another study found that exposure to peripubertal stress following fetal MIA exposure acted synergistically in activating hippocampal microglia as measured by CD68 and CD11b staining and increased levels of pro-inflammatory cytokines in the hippocampus of mice [110].

**Fig. 2:**
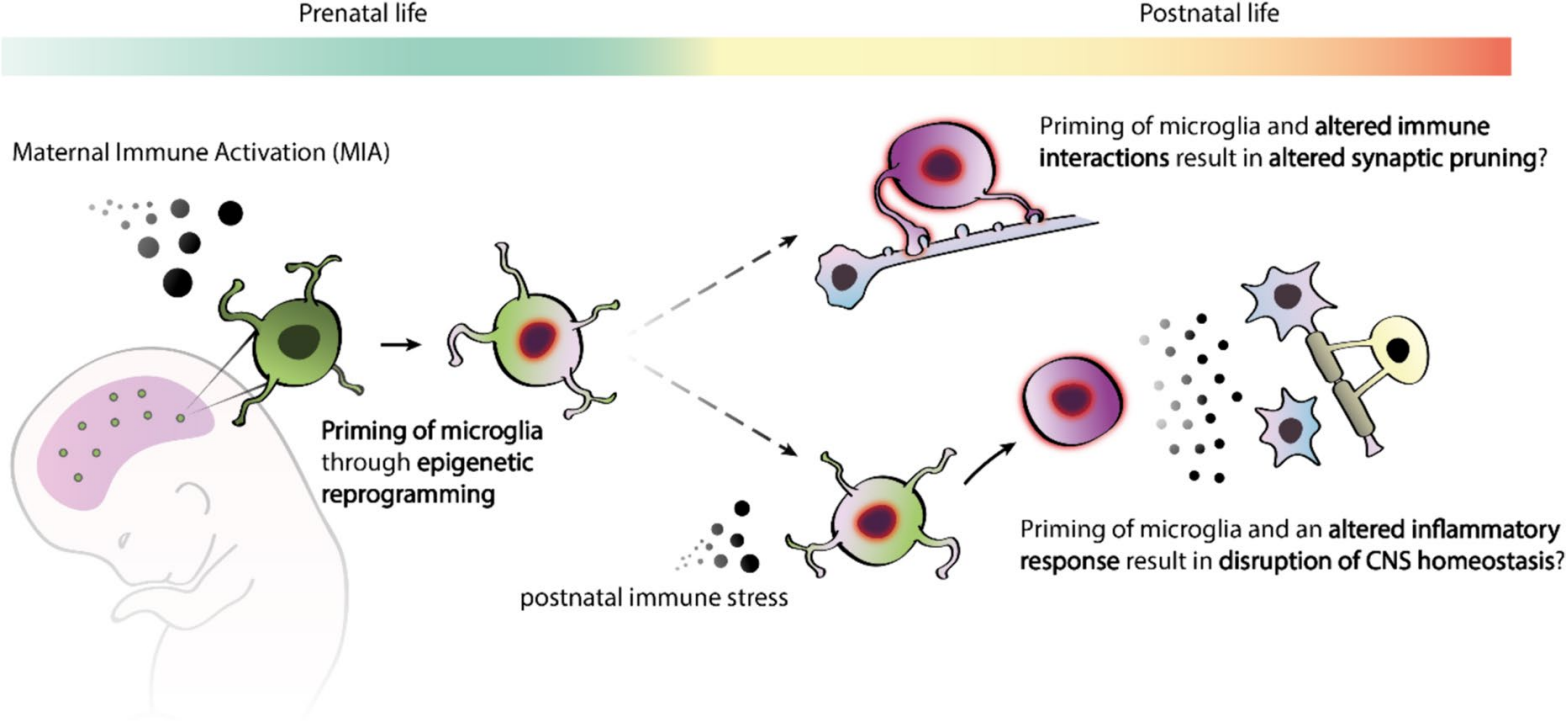
A hypothetical model illustrating maternal immune activation (MIA), microglial priming, and their impacts on microglial functioning in postnatal life. MIA leads to the 'priming' of microglia, which alters the microglial neuroinflammatory response upon re-exposure to an immune stressor later in life [75, 109], may be governed through epigenetic reprogramming. A disruption in the microglial immune response may i) impair microglia in maintaining central nervous system (CNS) homeostasis or ii) interfere with synaptic pruning in the postnatal brain, both of which rely on microglia-immune signalling

Overall, these studies indicate that effect of early life reprogramming of microglia can have significant functional consequences later in life. These can be unmasked upon restimulation, with signs of immune tolerance or training depending on the timing and mode of activation. Immune training or tolerance has already been shown to influence the development of neuropathology in animal models of Alzheimer's disease and stroke [107], as well as influence cognitive function in adults [75], demonstrating that immune priming of microglia can have a significant biological effect [107]. In a model for neurodevelopment, exposure to immune stress might act as a second hit for immune ‘primed’ microglia early in life, a phenomenon highly relevant for SCZ since manifestation of clinical symptoms typically occurs later in young adult life (Fig. 2). Microglial reprogramming results in an altered inflammatory response to immune stress which may disrupt CNS homeostasis. This is in line with studies showing a beneficial effect of minocycline, a drug that inhibits microglia, in MIA animal models [78, 111, 112] as well in patients with SCZ [113]. Alternatively, innate immune memory in fetal microglia may directly interfere with neurodevelopmental processes that require microglia and immune signalling (Fig. 2). For example, microglial-dependent synaptic pruning is highly reliant on the complement system [114].

It is currently not yet clear what constitutes the molecular mechanisms underlying the persistent change in the microglial transcriptome and the altered immune response in microglia following MIA. Epigenetic regulation in microglia, as observed innate immune memory, presents a potential candidate mechanism to explain the lasting effects of MIA. Similar to peripheral myeloid cells, microglia are capable of developing an innate immune memory through epigenetic programming [107, 115]. In a MIA mouse model, it has been demonstrated that the blunted immune response in adulthood was associated with changes in the microglial epigenetic landscape after LPS re-exposure, with more open-chromatin regions but less transcription factors occupying these regions [109]. A MIA mouse model of maternal allergic asthma also induces fetal neuroinflammation [116] and results in microglial-specific epigenetic changes, including sites associated with inflammatory genes overlapping with ASD risk annotated genes [117]. Immune activation early in postnatal life was also shown to change the microglial epigenetic landscape at gene promoter regions, and there was evidence suggestive of enhanced synaptic pruning and reduced dendritic spines, specifically in males [118] (Fig. 2). We propose that MIA exposure similarly reprograms the epigenetic signature of microglia in the fetal brain, leading to persistent changes in the microglial transcriptome and inflammatory response seen in postnatal life (Fig. 2). Intriguingly, repopulation of microglia using CSF1R inhibition has been successful in reversing MIA induced phenotypes [94, 109]. Following CSF1R inhibition, repopulation of the microglial cell pool comes from local self-renewal and is not dependent on the contribution of non-microglial cells [119]. On the transcriptomic level, initially these newborn microglia go from a ‘neonatal’ stage and gradually become more similar to adult [120]. On top of ameliorating the blunted immune response, repopulation of MIA-induced microglia did not show distinct clustering from control microglia (i.e. non-MIA and without CSF1R inhibition) [109]. Perhaps, these newly born microglia start without any pre-existing transcriptomic or epigenetic biases imprinted on them during development as a result from environmental disturbances such as MIA. In the future, it would be interesting to continue investigating microglial dynamics using CSF1R inhibition, especially in relation to changes in their epigenetic signature. For example, it is currently unclear whether the few surviving microglia following CSF1R inhibition are predetermined and if so, if this subpopulation of microglia has unique functional characteristics which are propagated to newborn cells after self-renewal. Conversely, if the surviving microglia following CSF1R inhibition are not unique, does the process of self-renewal initiate a transcriptomic reset, possibly through an epigenetic wipe, and thereby re-establishing the normal microglial transcriptomic and functional state. Further research is required to determine whether epigenetic mechanisms are responsible for the altered immune response following MIA induction.

## Conclusions

Currently it is not clear whether immune activation of microglia acts as the mediator driving the increased risk of NDDs when exposed to MIA. There is evidence, consisting mostly of animal studies, suggesting direct involvement of microglia in the MIA-induced effects observed at the neuronal-, particularly the PV interneurons, and synaptic level, both of which are strongly associated with the neuropathology of NDDs. There is also evidence that MIA has long-lasting functional consequences on the microglial inflammatory response, suggesting that neurodevelopmental- and/or CNS homeostatic processes requiring microglia and immune signalling are potentially compromised. How these effects are related to MIA induced NND-like behaviour, and ultimately how this translates to humans, remains to be understood. In order to better understand the role of microglia in relation MIA and NDDs, it is important to more comprehensively map the involvement of microglia in normal neurodevelopment. Recently, scRNA-seq and other transcriptomic studies revealed increased microglial heterogeneity during fetal brain development, identifying unique microglial subsets, hinting at functionally distinct roles. The appearance of DAM-like microglia, which were originally associated with neurodegenerative conditions, at different stages of development is particularly interesting since their phenotype suggests that these microglia are highly engaged. In the future, new tools such as spatial transcriptomics and high-plex spatial proteomics (i.e. imaging mass cytometry) can enhance our understanding of microglial heterogeneity in relation to other CNS cell types. This will enable us to map microglial-cell interactions at a single-cell level, providing deeper insight into their specific functional roles during neurodevelopment, and study alterations when exposed to MIA.

## Data Availability

No original data was generated for this review article.
